# The paralogous *SPX3* and *SPX5* genes redundantly modulate Pi homeostasis in rice

**DOI:** 10.1093/jxb/ert424

**Published:** 2013-12-24

**Authors:** Jing Shi, Han Hu, Keming Zhang, Wei Zhang, Yanan Yu, Zhongchang Wu, Ping Wu

**Affiliations:** State Key Laboratory of Plant Physiology and Biochemistry, College of Life Sciences, Zhejiang University, Hangzhou 310058, China

**Keywords:** Negative regulation, *Oryza sativa* L., Pi homeostasis, protein interaction, SPX proteins.

## Abstract

The importance of SPX-domain-containing proteins to phosphate (Pi) homeostasis and signalling transduction has been established in plants. In this study, phylogenetic analysis revealed that *OsSPX3* and *OsSPX5* (*SPX3/5*) are paralogous *SPX* genes (*SYG1/Pho81/XPR1*) in cereal crops. *SPX3/5* are specifically responsive to Pi starvation at both the transcriptional and post-transcriptional levels. Similar tissue expression patterns of the two genes and proteins were identified by *in situ* hybridization and the transgenic plants harbouring *SPX3pro-SPX3-GUS* or *SPX5pro-SPX5-GUS* fusions, respectively. Both SPX3/5 are localized in the nucleus and cytoplasm in rice protoplasts and plants. SPX3/5 negatively regulate root-to-shoot Pi translocation with redundant function. The data showed that the Pi-starvation-accumulated SPX3/5 proteins are players in restoring phosphate balance following phosphate starvation. *In vitro* and *in vivo* protein–protein interaction analyses indicated that these two proteins can form homodimers and heterodimers, also implying their functional redundancy. Genetic interaction analysis indicated that SPX3/5 are functional repressors of OsPHR2 (PHR2), the rice orthologue of the central regulator AtPHR1 for Pi homeostasis and Pi signalling. These results suggest that the evolution of the additional redundant paralogous *SPX* genes is beneficial to plants recovering Pi homeostasis after Pi starvation by PHR2 pathway.

## Introduction

The maintenance of phosphate (Pi) homeostasis in plants is crucial for plant growth and development. Pi homeostasis is determined by Pi acquisition through the root system, loading to the xylem, translocation between root and shoot, and remobilization of internal Pi, which is achieved by coordination of different Pi transporters underlying an elaborate Pi-signalling network comprising local and systemic machineries (see review by Chiou and Lin, 2011; Wu *et al.*, 2013).

The hydrophilic SPX domain (SYG1/Pho81/XPR1) is found at the N-termini of various proteins, particularly signal transduction proteins (Barabote *et al.*, 2006). Increasing evidence shows that the proteins containing the SPX domain are key players controlling a set of processes involved in Pi homeostasis (Hamburger *et al.*, 2002; Duan *et al.*, 2008; Wang *et al.*, 2009a; Lin *et al.*, 2010; Liu *et al*., 2010, 2012; Kant *et al.*, 2011). In plants, SPX-domain-containing proteins can be divided into four classes depending on the presence of additional protein domains. Proteins exclusively harbouring the SPX domain are referred to as SPX proteins and fall into class I, with four members in *Arabidopsis* and six members in rice (Duan *et al.*, 2008; Secco *et al.*, 2012). Phylogenetic analysis has revealed three evolutionary clades of these SPX proteins: clade I contains SPX1 and SPX2, clade III contains SPX4, and clade II contains SPX3 in *Arabidopsis* (AtSPX3) and three paralogous proteins in rice, designated SPX3, SPX5, and SPX6 (Secco *et al.*, 2012).

Transcriptional and histochemical analyses have shown that all of the *SPX* genes, with the exception of *SPX4*, are responsive to Pi starvation (Duan *et al.*, 2008; Wang *et al.*, 2009b). The various subcellular localizations of the SPX proteins in *Arabidopsis* and rice have been described (Duan *et al.*, 2008; Wang *et al.*, 2009b), with results suggesting that the different SPX proteins may play distinct roles in Pi signalling and homeostasis processes.

In *Arabidopsis*, the expression of *AtSPX1–4* genes is reduced to different extents in the *phr1* mutant under Pi starvation (Duan *et al.*, 2008), indicating that these genes are downstream of the central regulator AtPHR1 for Pi signalling. Overexpression of *AtSPX1* upregulates Pi-starvation-induced (PSI) genes, such as *ACP5*, *PAP2*, and *RNS1*, suggesting a potential transcriptional regulation role of AtSPX1 in Pi starvation. Repression of *AtSPX3* alters the response of PSI genes to Pi starvation, resulting in total phosphorus higher in shoots and lower in roots (Duan *et al.*, 2008).

In rice, OsSPX1 (SPX1), similar to AtSPX3, is involved in negative regulation to adjust the expression of several PSI genes under Pi-limited conditions (Wang *et al.*, 2009a). Genetic analysis has demonstrated that SPX1 counteracts the function of OsPHR2 (PHR2), a rice orthologue of AtPHR1 as a central Pi-signalling regulator (Rubio *et al.*, 2001; Zhou *et al.*, 2008; Liu *et al.*, 2010), inducing expression of the low-affinity Pi transporter gene *PT2*, which plays a major role in Pi translocation and accumulation (Ai *et al.*, 2009). The function of other SPX proteins in rice, however, has not been yet characterized.

In this study, a phylogenetic tree was generated from alignments of the protein sequences of all of the SPX proteins in representative dicotyledonous plants and cereal monocotyledonous crops. The results show that three paralogous SPX proteins, SPX3, SPX5, and SPX6, have evolved in cereal crops. The data reveal that SPX3 and SPX5 can form homodimers and/or heterodimers. SPX3 and SPX5 play roles in maintaining cellular Pi homeostasis when plants are exposed to an external change in Pi, and this implies a more controllable regulation system for crops to adapt to environmental Pi variations. In addition, genetic interaction analyses indicate that SPX3 and SPX5 are repressors of PHR2 function. This finding increases the understanding of the integrated regulation of SPX domain proteins in Pi homeostasis and signalling in plants.

## Materials and methods

### Plant materials and growth conditions

The *spx3* T-DNA insertional mutant (ALNE05) (japonica cv. Nipponbare) was obtained from the CIRAD database (Centre de Coopération International en Recherche Agronomique pour le Développement; http://orygenesdb.cirad.fr). The *spx3* T-DNA insertion site at the second exon was determined by sequencing analysis (Supplementary Fig. S3, available at *JXB* online). The primers for the identification of the *spx3* mutant are listed in Supplementary Table S1. The hydroponic experiments were performed using rice solution culture (Zhou *et al.*, 2008). Rice plants were grown in a greenhouse with a 12/12 light/dark cycle (200mol m^–2^ s^–1^ photon density) at 30/22 °C after germination. Humidity was controlled at approximately 60%.

### Cloning of *SPX5* and *SPX6*

Total RNA was extracted from the rice seedling roots after 7 d of Pi-deficient treatment using TRIzol Reagent (Invitrogen, Carlsbad, CA, USA). SMART RACE cDNA Amplification Kit (Clontech, USA) was used for rapid amplification of cDNA ends (RACE); the primers for RACE-PCR are provided in Supplementary Table S1. The PCR products were gel purified and subcloned into the pGEM-T Easy vector (Promega) for sequencing.

### Construction of the phylogenetic tree

Amino acid sequences of the tested species SPX proteins were retrieved from GenBank (http://www.ncbi.nlm.nih.gov/Genbank). The phylogenetic tree was constructed using MEGA 5.10 (Tamura *et al.*, 2011) based on the neighbour-joining method with parameters of Poisson correction model, pairwise deletion, and bootstrap (1000 replicates; random seed).

### *In situ* hybridization and GUS histochemical analyses

*In situ* hybridization was performed as described previously (Zhao *et al.*, 2009). The primer pairs SPX3 insitu-F/SPX3 insitu-T7-R, and SPX5 insitu-F/SPX5 insitu-T7-R were used to amplify the antisense templates of *SPX3* and *SPX5*. The primers SPX3 insitu-SP6-F/SPX3 insitu-R, and SPX5 insitu-SP6-F/SPX5 insitu-R were used to amplify the sense templates of *SPX3* and *SPX5*. For development of SPX3pro-SPX3-GUS and SPX5pro-SPX5-GUS transgenic plants, the Nipponbare genomic DNA fragments containing the coding region of *SPX3* or *SPX5* and approximately 2.9-kb promoter sequence were amplified with primer pairs SPX3-GUS-F/SPX3-GUS-R and SPX5 -GUS-F/SPX5-GUS-R, and cloned into the binary vector GUS-pBI101.3 by In-fusion kit (Clontech), respectively. The GUS constructs were introduced into rice plants (Nipponbare) using the *Agrobacterium*-mediated transformation. Histochemical GUS analysis was performed as described previously (Jefferson *et al*., 1987). The primers are listed in Supplementary Table S1.

### Development of the transgenic plants

The transgenic plant with knockdown of *SPX5* was developed using RNA interference (RNAi). A 260-bp fragment of *SPX5* in sense and antisense orientations was inserted into both sides of the second intron of the maize *NIR1* gene, and the fragment was subcloned into the 35S-pCAMBIA1300-mod vector. For development of transgenic plants with overexpression of *SPX3* and *SPX5* (*SPX3*/5), full-length *SPX3*/5 were cloned into the 35S-pCAMBIA1300-mod vector. These constructs were transformed into rice using the *Agrobacterium*-mediated transformation. The primers are listed in Supplementary Table S1.

### Yeast two-hybrid assay

The yeast two-hybrid assay was performed using the Matchmaker GAL4 Two-Hybrid System3 (Clontech), following the manufacturer’s protocol. To construct SPX3-pGADT7 (AD-SPX3), SPX3-pGBKT7 (BD-SPX3), AD-SPX5, and BD-SPX5 vectors, full-length coding sequences of *SPX3* and *SPX5* were amplified by primer pairs SPX3-YF/SPX3-YR and SPX5-YF/SPX5-YR with adapters (Supplementary Table S1) for *Nde*I-*Bam*HI and *Nde*I-*Eco*RI digestion sites, and cloned into pGBKT7 and pGADT7, respectively. The vectors were cotransformed into yeast strain AH109 with the resulting constructs and plated onto synthetic medium lacking Trp, Leu, His and Ade (SD/–Trp–Leu–His–Ade) or SD/–Trp–Leu. Plates were photographed after incubation at 30 °C for 3–5 d.

### Coimmunoprecipitation assay

To construct the 35S-SPX3-MYC, 35S-SPX5-MYC, 35S-SPX3-FLAG, and 35S-SPX5-FLAG vectors, full-length coding sequences of *SPX3* and SPX5 were amplified by primer pairs SPX3-MYC-F/SPX3-MYC-R, SPX5-MYC-F/SPX5-MYC-R, SPX3-FLAG-F/SPX3-FLAG-R, and SPX5-FLAG-F/SPX5-FLAG-R (Supplementary Table S1), and were inserted into the modified pCAMBIA1300–35S-MYC vector (Hong *et al.*, 2012) or 35S-FLAG vector (Menon *et al.*, 2005), respectively. The resulting constructs were transiently expressed in tobacco leaves (*Nicotiana benthamiana*) by *Agrobacterium tumefaciens* EHA105 infiltration. The leaves were harvested after 2 d and lysed with a buffer (50mM Tris, 50mM NaCl, 1mM EDTA, 1mM DTT, 1mM PMSF, 100 µM MG132 (Sigma-Aldrich), and 1× protease inhibitor cocktail (Roche)). Lysates were incubated for 4h with anti-FLAG antibody-conjugated beads (Sigma) at 4 °C. The beads were then washed three times and solubilized in 15 µl SDS sample buffer. Samples were analysed by 12% SDS-PAGE and immunoblotted as described in immunoblot analyses. Each immunoblot was incubated with the appropriate primary antibody (1:250; mouse anti-FLAG monoclonal or mouse anti-MYC monoclonal, Sigma) in 1× TBST for overnight at 4 °C. Membranes were developed using peroxidase-conjugated secondary antibody (anti-mouse IgG, Sigma), and proteins were detected by chemiluminescence using an ECL-detecting reagent according to the manufacturer’s protocol (Thermo Scientific).

### Bimolecular fluorescence complementation assays

For the production of bimolecular fluorescence complementation (BiFC) vectors, the full-length coding sequences of *OsSPX3/5* were amplified using the primer pairs SPX3–2YN-F/SPX3–2YN-R and SPX3–2YN-F/SPX3–2YN-R (Supplementary Table S1), and cloned into p2YN or p2YC as a fusion with the N-terminal or C-terminal fragment of YFP, resulting in SPX3-YFP^N^, SPX3-YFP^C^, SPX5-YFP^N^, and SPX5-YFP^C^. The resulting constructs were transiently expressed in tobacco leaves by *A. tumefaciens* EHA105 infiltration. BiFC experiments were performed as described previously (Yang *et al.*, 2007). YFP fluorescence was observed and photographed by confocal microscopy (LSM 510 META, Zeiss, Germany) at 48–72h after infiltration.

### RNA isolation, reverse-transcription PCR, and qRT-PCR

RNA isolation and quantitative real-time PCR (qRT-PCR) were performed as described previously (Liu *et al.*, 2010). qRT-PCR for quantification of mature miR399 was performed following a published protocol (Varkonyi-Gasic *et al.*, 2007). The primers for reverse-transcription PCR and qRT-PCR analyses are listed in Supplementary Table S1.

### Measurements of Pi concentration and Pi uptake ability in plants

Measurement of Pi concentration, biomass, Pi uptake ability, and distribution in plants were performed as described previously (Zhou *et al.*, 2008; Wu *et al.*, 2011).

## Results

### *SPX3*, *SPX5*, and *SPX6* are paralogous genes in crops

**Fig. 1.**
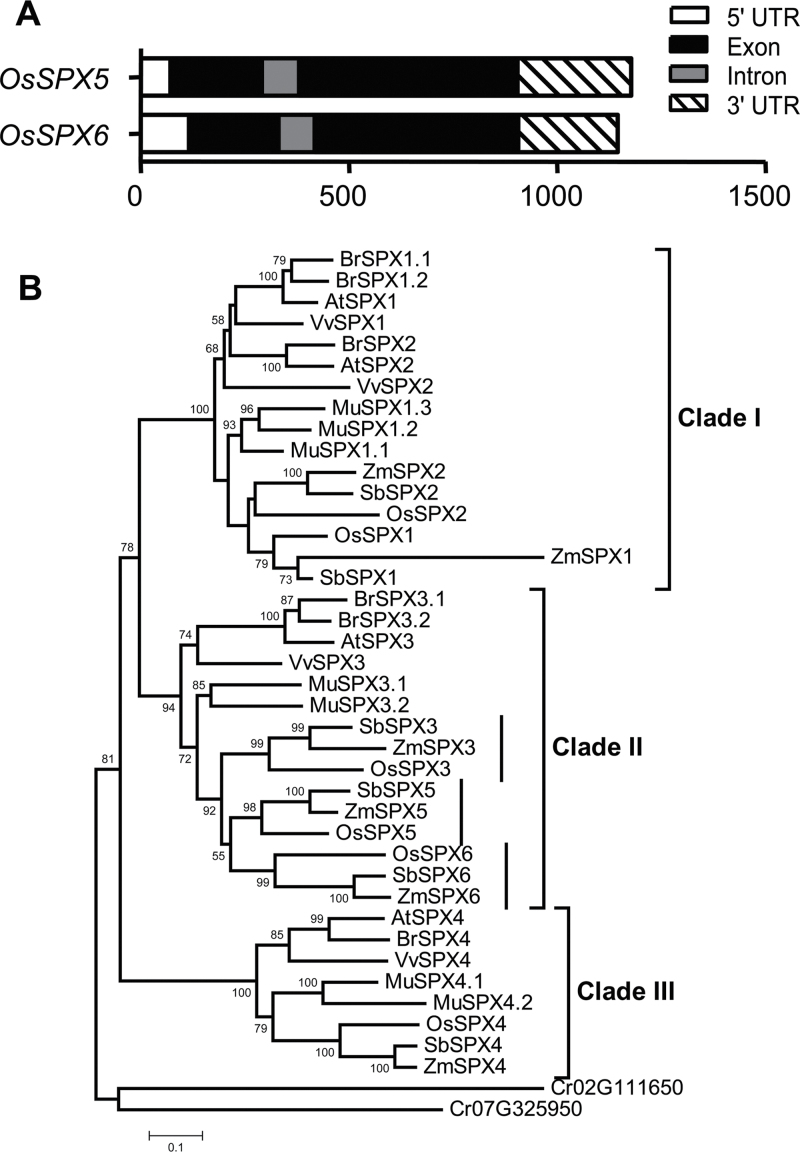
Structures of *SPX5* and *SPX6*, and phylogenetic relationships of SPX proteins in dicotyledons and monocotyledons. (A) Structures of *SPX5* and *SPX6* determined by RACE assays. (B) Root tree constructed using MEGA 5.1 by the neighbour-joining method with bootstrap probabilities based on 1000 replicates shown at branch nodes. At, *Arabidopsis thaliana*; Br, Chinese cabbage (*Brassica rapa*); Cr, *Chlamydomonas reinhardtii* (outgroup); Mu, banana (*Musa acuminata*); Os, rice (*Oryza sativa*); Sb, sorghum (*Sorghum bicolor*); Vv, grape (*Vitis vinifera*); Zm, maize (*Zea mays*).

Six *SPX* genes (*SPX1–6*) have been identified in rice that are induced by Pi starvation with the exception of *SPX4* (Wang *et al.*, 2009b). The current data further showed that *SPX3*, *SPX5*, and *SPX6* are specifically induced by Pi starvation, which was remarkably reduced in the *phr2* mutant (Supplementary Fig. S1; Chen *et al.*, 2011). The full-length cDNA sequences of *SPX1–4* are available in the Rice Genome database (http://rice.plantbiology.msu.edu/). To generate a rooted phylogenetic tree for all of the SPX proteins, full-length cDNA of *SPX5* and *SPX6* was cloned using RACE assays based on the conserved SPX domain sequence in the rice genome (Rice Genome Annotation Project). The deduced full-length cDNA sequence of *SPX5* (GenBank accession number KF267997) consists of 1090 nucleotides, containing an open reading frame of 744-bp for 247 amino acids, a 72-bp 5′-untranslated region, and 274-bp 3′-untranslated region; *SPX6* (GenBank accession number KF267998) consists of 1045 nucleotides, containing an open reading frame of 705-bp for 234 amino acids, a 116-bp 5′-untranslated region, and a 239-bp 3′-untranslated region. The gene structure analysis showed two exons and one intron in *SPX5* and *SPX6*, respectively (Fig. 1A).

A phylogenetic tree was generated from alignments of the sequences of the six SPX proteins in dicotyledonous plants, monocotyledonous cereal crops, and the ancestral genome *Chlamydomonas reinhardtii*. The dicotyledonous plants included *Arabidopsis*, Chinese cabbage (*Brassica rapa*), and grape (*Vitis vinifera*), and monocotyledonous cereal crops included rice (*Oryza sativa* L.), maize (*Zea mays*), and sorghum (*Sorghum bicolor*). Banana (*Musa acuminate*), a monocotyledonous herb of the order Zingiberales and a sister group to the well-studied Poales including cereals (D’Hont *et al.*, 2012), was also analysed. The phylogenetic analysis showed that the six SPX proteins fall into three clades (Fig. 1B). Clades I and III contain SPX1 and SPX2, and SPX4, respectively, with a more divergent evolutionary relationship between dicotyledonous and monocotyledonous plants. Clade II contains SPX3, SPX5, and SPX6 proteins, which fall into three subgroups in monocotyledonous crops, while SPX3 is only present in the dicotyledonous plants *Arabidopsis* and grape. Chinese cabbage and banana contain two copies of SPX3 as sister pairs. The results indicate that the three paralogous genes (*SPX3*, *SPX5*, and *SPX6*) are evolved in cereal crops.

### Subcellular localization and expression patterns of SPX3 and SPX5

This work examined the subcellular localization of the paralogous proteins in rice protoplasts and rice plants. The similar subcellular localizations of SPX3 and SPX5 (SPX3/5) in both nucleus and cytoplasm were visualized using the fusions of SPX3-GFP and SPX5-GFP (Supplementary Fig. S2). Because of the same subcellular localization of SPX3/5, this study focused on the functional relationship of these two SPX proteins.

**Fig. 2.**
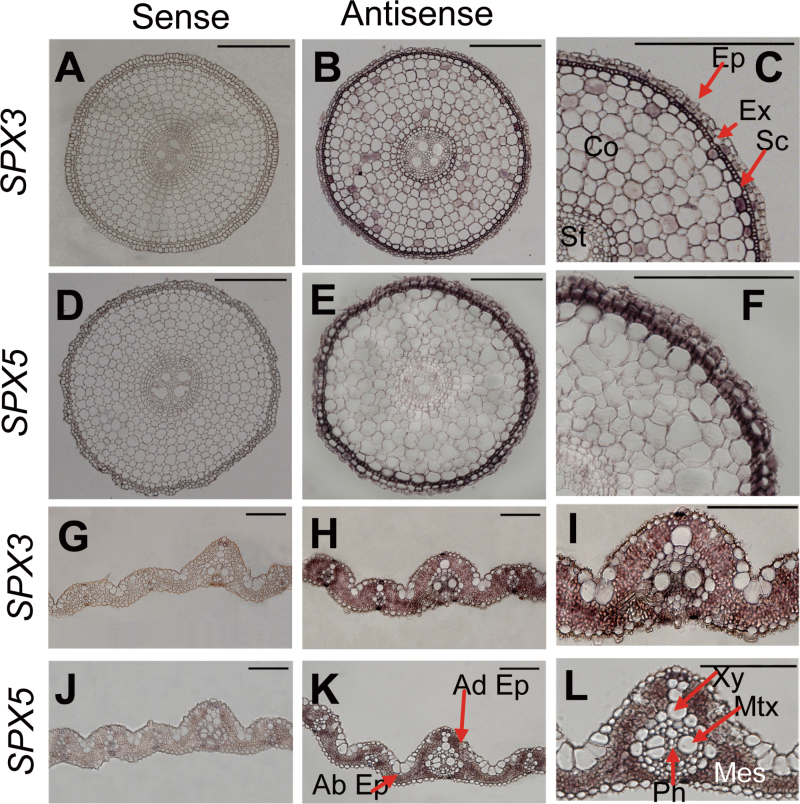
Expression patterns of *SPX3/5* in roots and leaf blades indicated by *in situ* hybridization. (A–F) *In situ* hybridization on cross-sections of primary roots with *SPX3* or *SPX5* antisense probes (B and E); negative control with sense probes (A and D); and detailed antisense signals (C and F). (G–L) *In situ* hybridization on cross-sections of leaf blades with *SPX3* or *SPX5* antisense probes (H and K); negative control with sense probes (G and J); and detailed antisense (I and L). Roots and leaves were sampled from 20-d-old seedlings treated with Pi starvation for 7 d. Ab, abaxial; Ad, adaxial; Co, cortex; Ep, epidermis; Ex, exodermis; Mes, mesophyll; Mtx, metaxylem; Ph, phloem; Sc, sclerenchyma layer; St, stele; Xy, xylem. Bars, 100 µm.

The tissue expression patterns of SPX3/5 were investigated using *in situ* hybridization with probes specific to SPX3 or SPX5 (Supplementary Table S1). The roots of 15-d-old plants treated with Pi starvation for 7 d had an overlapped hybridization signal of SPX3/5 in root epidermis, exodermis, and the sclerenchymal layer. The hybridization signal was also detected for SPX3 in cortex and endosperm (Fig. 2A–F). Under Pi starvation, the specific hybridization signals of SPX3/5 were present in mesophyll and phloem in the vascular bundles (Fig. 2G–L). The signal was highly specific and did not appear in leaf and root tissues when using control sense probes.

**Fig. 3.**
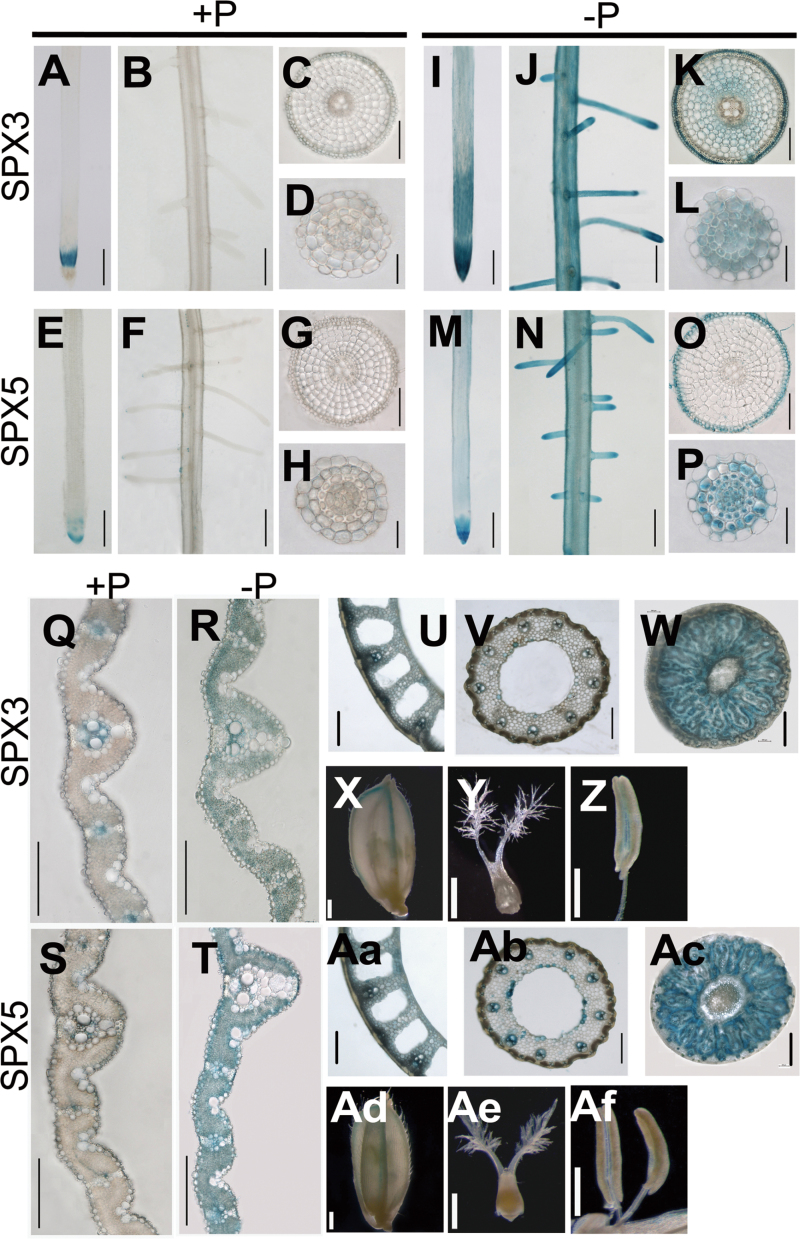
Tissue expression patterns of SPX3 and SPX5 indicated by expression *SPX3pro*-*SPX3-GUS* and *SPX5pro*-*SPX5-GUS* fusions in the transgenic plants. (A–P) SPX3 and SPX5 expression in root tips (A, E, I, M) and lateral roots (B, F, J, N) under +P (200 µM Pi) or –P conditions (0 µM Pi); bars, 200 µm and SPX3 and SPX5 expression in cross-sections of primary roots (C, G, K, O) and lateral roots (D, H, L, P) under +P or –P conditions; bar, 20 µm. (Q–T) SPX3 and SPX5 expression patterns in cross-section of leaf blade under +P (Q, S) or –P (R, T) conditions; bar, 20 µm. (U–Af) GUS staining on hull (X, Ad), stigma (Y, Ae), anther (Z, Af), and cross-sections of leaf sheath (U, Aa), stem (V, Ab), and node (W, Ac). Bars, 50 µm (U–Z, Aa, Ad–Af; 400 µm (V, W, Ab, Ac).

To determine the expression patterns of SPX3/5 proteins, this work developed transgenic plants harbouring the *SPX3pro-SPX3-GUS* or *SPX5pro-SPX5-GUS* fused gene. Histochemical analyses of GUS activity in the transgenic plants grown under Pi-supplied (200 µM Pi) and Pi-deficient conditions showed that SPX3/5 were induced under deficient Pi with consistent patterns of their transcripts, as indicated by *in situ* hybridization. The same expression patterns of SPX3/5 were observed in roots and leaf blades (Fig. 3A–T). GUS activity was also detected in leaf sheath, node, stem vascular bundle, hull, and anther (Fig. 3U–Af). The same general expression patterns of SPX3/5 suggest that they may have similar functions.

### SPX3/5 redundantly modulate Pi homeostasis

**Fig. 4.**
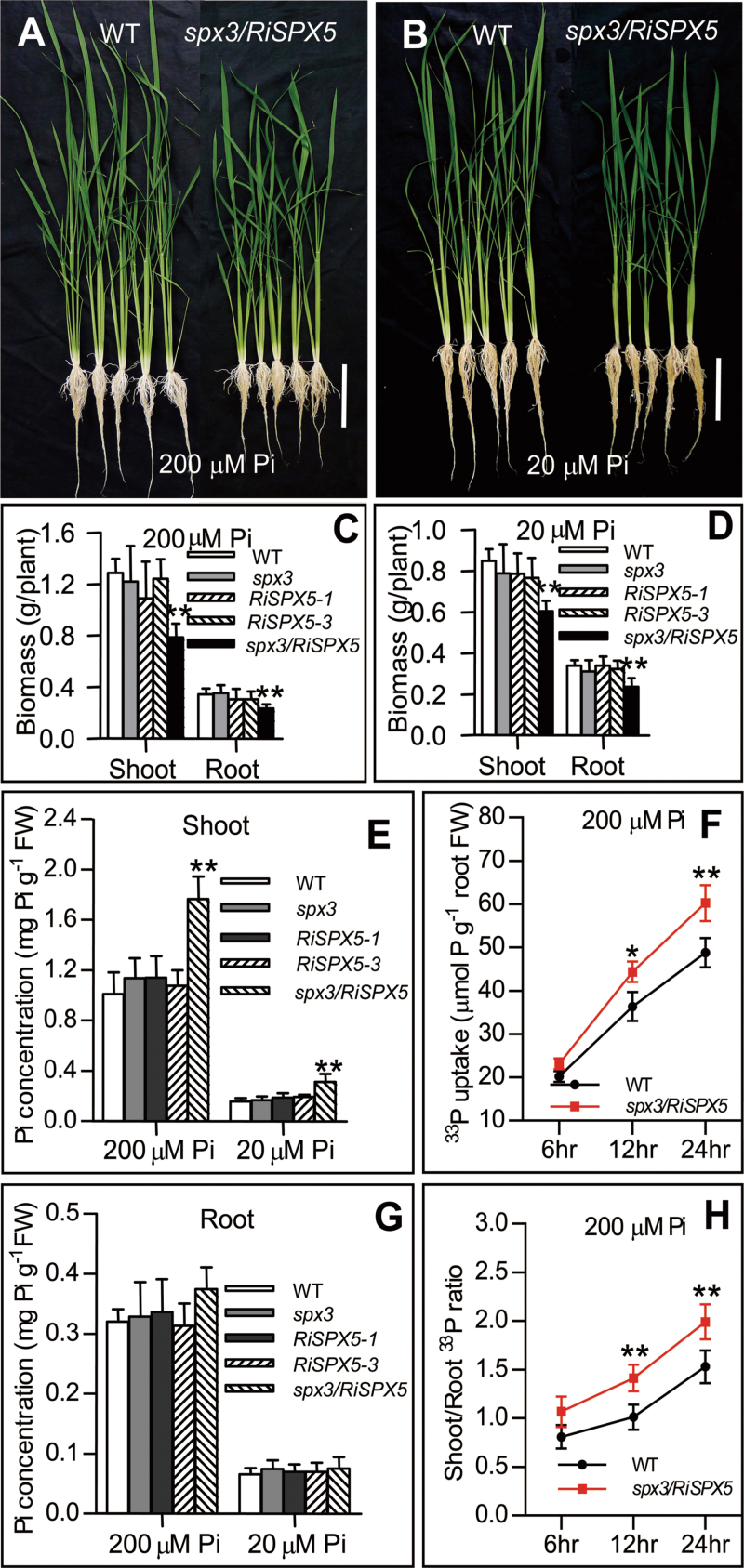
SPX3 and SPX5 redundantly function on Pi homeostasis. (A and B) Phenotypic performances of 30-d-old wild-type (WT) and *spx3/RiSPX5* plants under 200 µM Pi (A) and 20 µM Pi (B) conditions; bar, 20cm. (C and D) Dried biomass of 30-d-old WT, *spx3*, *RiSPX5-1*, *RiSPX5-3*, and *spx3/RiSPX5* plants. (E and G) Cellular Pi concentration in the shoots (E) and roots (G) of WT, *spx3*, *RiSPX5-1*, *RiSPX5-3*, and *spx3/RiSPX5* plants grown under 200 or 20 µM Pi conditions. (F) Uptake rate of [^33^P] Pi in WT and *spx3*/*RiSPX5* plants. (H) The shoot-to-root ratio of [^33^P] Pi concentration of WT and *spx3*/*RiSPX5* plants. Plants were supplied with ^33^P-labelled Pi (H_3_^33^PO_4_) for 6, 12, and 24h. Values represent mean ± standard deviation of five biological replicates. Data significantly different from the corresponding wild-type controls are indicated *P < 0.05; (**P < 0.01, Student’s t-test).

This work isolated a *spx3* mutant (Nipponbare, ALNE05) from the CIRAD T-DNA insertion library (Supplementary Fig. S3A–C). Because the *spx5* mutant is unavailable, the transgenic plants (Nipponbare) with repression of *SPX5* were developed using RNAi (Supplementary Fig. S3D, G). The plants with repression of *SPX5* (*RiSPX5*) under the *spx3* mutant background were developed by a cross between the homozygous *spx3* mutant and plants harbouring a single copy of a plasmid containing the *SPX5-RNAi* vector (designated *spx3/RiSPX5*). The wild-type plants (Nipponbare), *spx3* mutants, two independent lines of *RiSPX5* plants (*RiSPX5-1* and *RiSPX5-3*), and *spx3/RiSPX5* plants were used for cellular Pi concentration and Pi signalling analyses in hydroponic cultures. Under both high Pi (200 µM Pi) and low Pi (20 µM Pi) conditions, no significant phenotypic difference between wild-type plants and *spx3* mutants or *RiSPX5* plants was observed (data not shown), while the growth of *spx3/RiSPX5* plants was significantly inhibited compared to wild-type plants (Fig. 4A–D). A significantly higher shoot Pi concentration was observed in *spx3/RiSPX5* plants compared to wild-type plants, but not in *spx3* mutants or *RiSPX5* plants (Fig. 4E). No statistically significant difference was observed in root Pi concentration between wild-type and *spx3/RiSPX5* plants under either Pi condition (Fig. 4G). The significantly higher Pi-uptake ability and shoot-to-root ratio of Pi in *spx3/RiSPX5* plants compared to wild-type plants were confirmed by ^33^P-labelled Pi uptake and concentration ratio of shoots to roots (Fig. 4F, H). These results indicated the redundant negative effect of SPX3 and SPX5 on Pi homeostasis.

### SPX3 and SPX5 negatively regulate Pi signalling

**Fig. 5.**
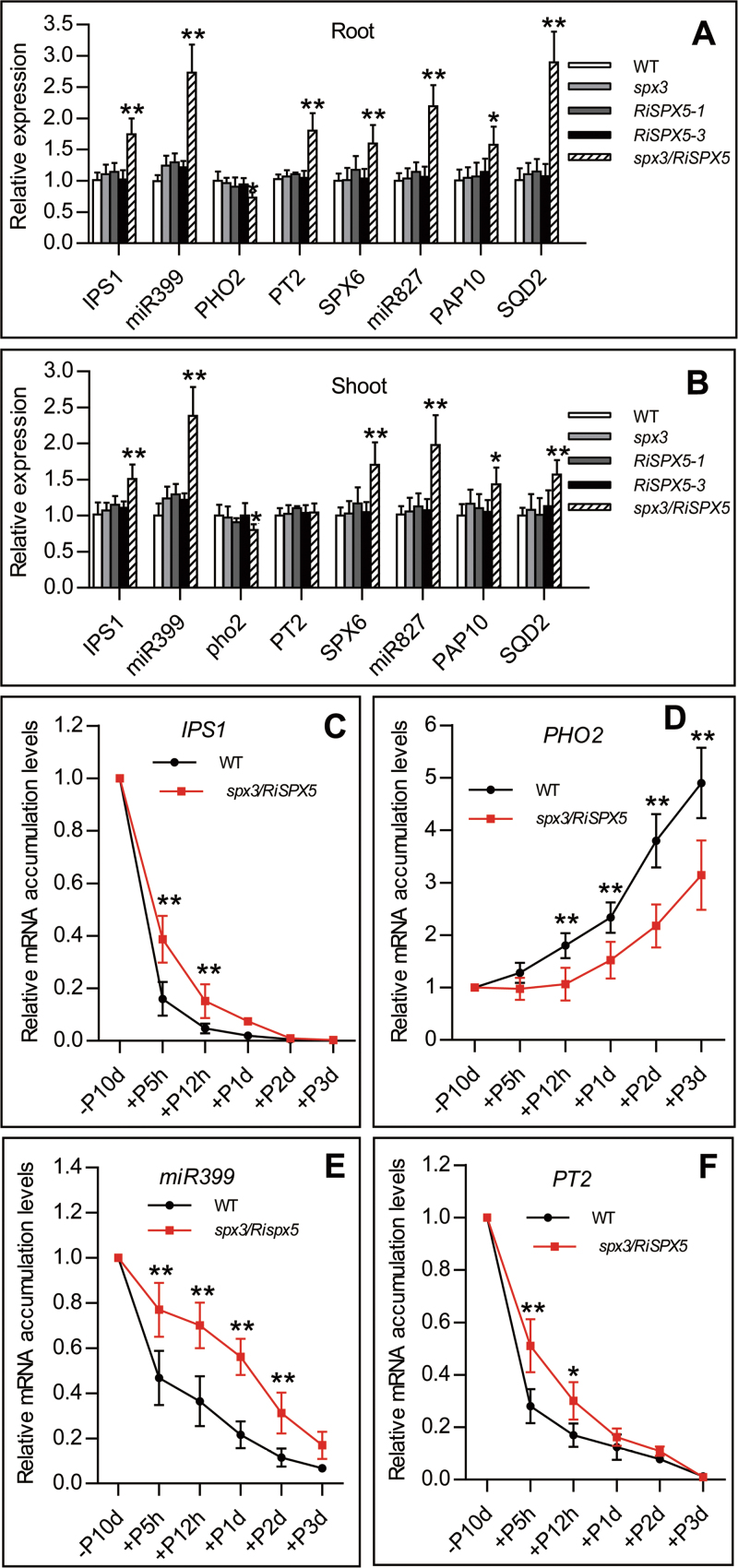
SPX3/5 proteins negatively regulate Pi signalling. (A and B) qRT-PCR analysis for transcript accumulation levels of the Pi-starvation-responsive genes downstream of *PHR2* in the wild-type (WT), *spx3* mutant, two independent lines of *RiSPX5* (*RiSPX5-1* and *RiSPX5-3*) and *spx3/RiSPX5* plants under Pi-supplied (200 µM Pi) condition. (C–F) qRT-PCR analysis for transcript accumulation levels of Pi-starvation-responsive genes in the roots of 20-d-old WT and *spx3*/*RiSPX5-3* plants over 3 d of Pi recovery after Pi starvation for 10 d. Data are ratio of the signal at a given time to the signal at Pi starvation point. Values represent mean ± standard deviation of three biological replicates. Data significantly different from the corresponding wild-type controls are indicated (*P < 0.05, **P < 0.01, Student’s t-test).

The redundant negative effect of SPX3 and SPX5 on Pi signalling was indicated by the expression of the Pi-starvation-responsive genes *IPS1*, *miR399*, *PT2*, *PHO2*, *SPX6*, *miR827*, *PAP10*, and *SQD2*. Significantly different transcript accumulation levels of the tested genes in *spx3/RiSPX5* plants grown under Pi-sufficient (200 µM Pi) condition were detected compared with wild-type, *spx3* mutant, and *RiSPX5* plants (Fig. 5A, B). Under Pi deficiency for 10 d, however, no significant difference in Pi signalling between wild-type and *spx3/RiSPX5* plants was observed (data not shown). Given that both transcripts and proteins of *SPX3/5* are induced by Pi starvation and that induced protein levels were maintained for a longer time than the induced transcript levels by recovery of Pi after Pi starvation (Supplementary Fig. S4), it was hypothesized that the induced proteins might play a role in Pi signalling. To investigate this possibility, this work tested the reduction of Pi-starvation signalling in a Pi-recovery time course after Pi starvation for 10 d. The elapsed time of reduction of Pi-starvation signalling after Pi recovery in *spx3/RiSPX5-3* plants was prolonged compared to wild-type plants in roots (Fig. 5C–F), and the similar trend was observed in shoots (data not shown), confirming that SPX3/5 proteins are involved in a negative regulation of Pi signalling.

### SPX3 and SPX5 are repressors of function of PHR2

**Fig. 6.**
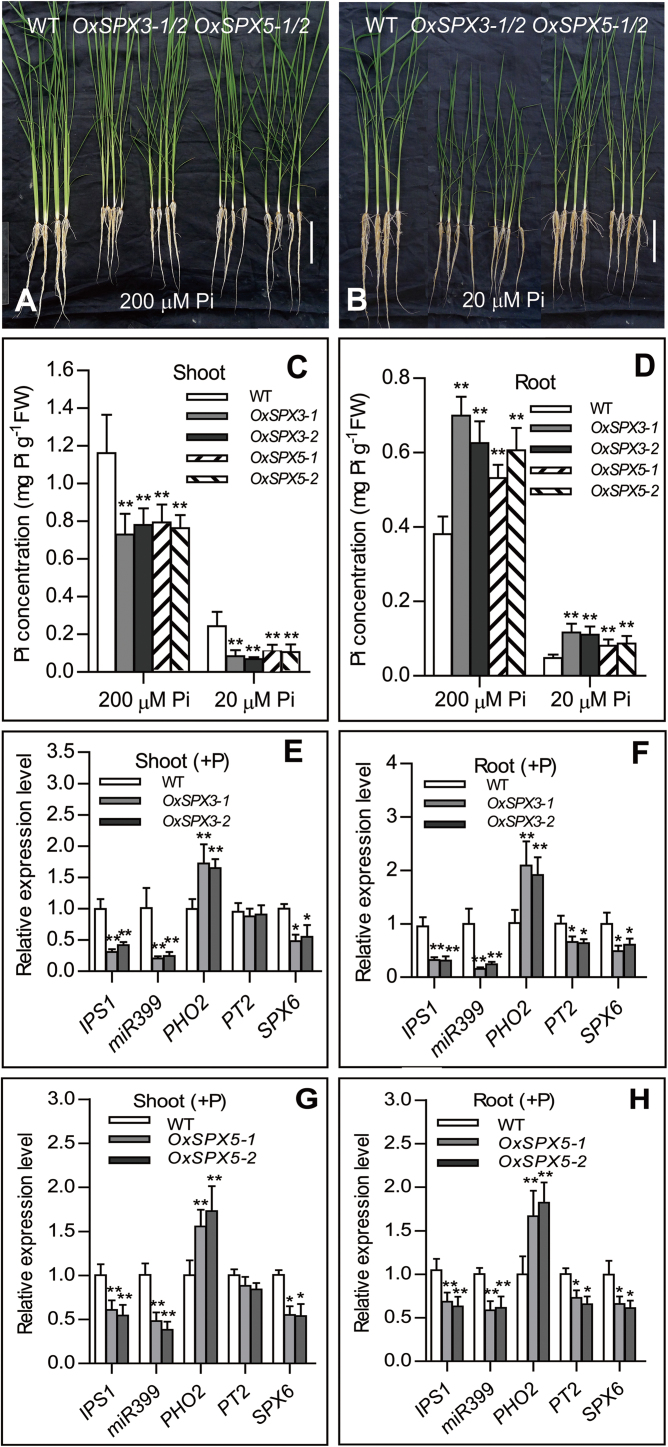
Overexpression of *SPX3* and *SPX5* impede root-to-shoot Pi translocation. (A and B) Phenotype of the wild-type (WT) and transgenic plants with overexpressed *SPX3* (OxSPX3) and *SPX5* (OxSPX5) under 200 µM Pi (A) and 20 µM Pi (B) conditions for 30 d; bar, 20cm. (C and D) Cellular Pi concentration in shoots (C) and roots (D) of WT, OxSPX3, and OxSPX5 plants grown under 200 µM or 20 µM Pi conditions. (E–H) qRT-PCR analysis for transcript accumulation levels of Pi-starvation-responsive genes in shoots and roots of WT, OxSPX3-1/2 (E and F) and OxSPX5-1/2 (G and H) plants under Pi-supplied (+P) condition (200 µM Pi). Values represent mean ± standard deviation of three biological replicates. Data significantly different from the corresponding wild-type controls are indicated (*P < 0.05, **P < 0.01, Student’s t-test).

Previous reports have demonstrated that SPX1 is a repressor of PHR2 function (Wang *et al.*, 2009a; Liu *et al.*, 2010). To determine whether SPX3/5 have the similar function, this work first developed SPX3-overexpressing and SPX5-overexpressing plants. Two independent lines with overexpression of SPX3 or SPX5, as confirmed by Southern blotting (Supplementary Fig. S3E, F, and H), were used for the hydroponic experiments with high Pi (200 µM Pi) and low Pi (20 µM Pi) levels. Similar to SPX3 overexpressors, overexpression of SPX5 inhibited plant growth under both Pi levels (Fig. 6A, B; Supplementary Fig. S5; Wang *et al.*, 2009b). Significantly lower shoot and higher root Pi concentrations were observed in the overexpressors compared with the wild-type plants under both Pi levels (Fig. 6C, D). These results indicate the similar function of SPX3 and SPX5 in root-to-shoot Pi translocation. Then, the transcript accumulation levels of these PSI genes downstream of PHR2 were tested in the plants with overexpressed SPX3/5. Compared with wild-type plants, a significant reduction of IPS1, miR399, PT2, and SPX6, and increase of PHO2 in shoots and roots under the Pi-supplied conditions were observed (Fig. 6E–H).

**Fig. 7.**
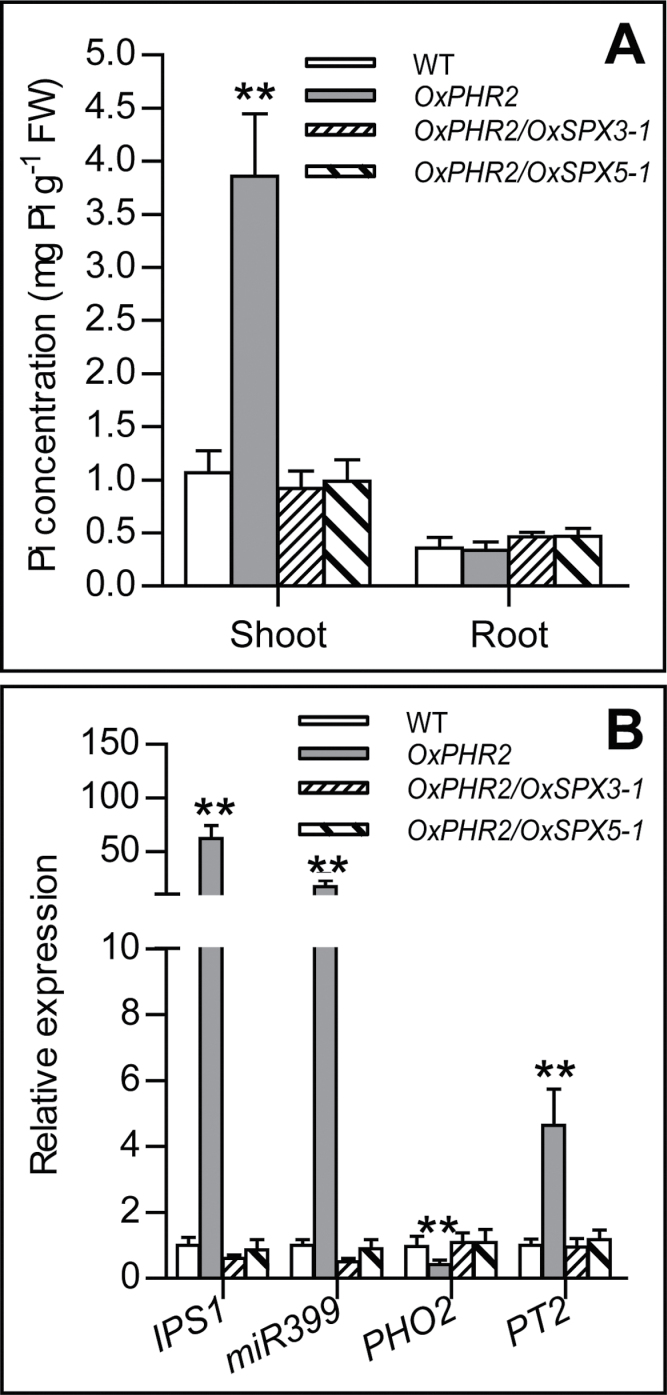
SPX3 and SPX5 are repressors of PHR2. (A) Pi concentration of the wild-type (WT), OxPHR2, and double overexpressed plants OxPHR2/OxSPX3 and OxPHR2/OxSPX5. The plants grown in solution culture with 200 µM Pi and 30-d-old plants were used for the analysis. Values represent mean ± standard deviation of five biological replicates. (B) qRT-PCR analysis for transcript accumulation levels of Pi-starvation-responsive genes in the roots of 14-d-old WT, OxPHR2, OxPHR2/OxSPX3, and OxPHR2/OxSPX5 plants. Values represent mean ± standard deviation of three biological replicates. Data significantly different from the corresponding wild-type controls are indicated (**P < 0.01, Student’s t-test).

This work developed plants simultaneously overexpressing SPX3 and PHR2 or SPX5 and PHR2 through crossing between the plants with overexpressed PHR2 (Zhou *et al.*, 2008) and overexpressed SPX3 or SPX5 plants (designated OxPHR2/OxSPX3 and OxPHR2/OxSPX5). As with SPX1, overexpression of SPX3 or SPX5 completely reduced excessive Pi accumulation in the shoots (Fig. 7A). The upregulation of IPS1, miR399, and PT2, and the reduction of PHO2 driven by PHR2 overexpression, was repressed by overexpression of SPX3/5 in roots (Fig. 7B), and the similar results were observed in shoots (data not shown). The results indicated that SPX3/5 are the repressors of PHR2.

### Protein interaction between SPX3 and SPX5

**Fig. 8.**
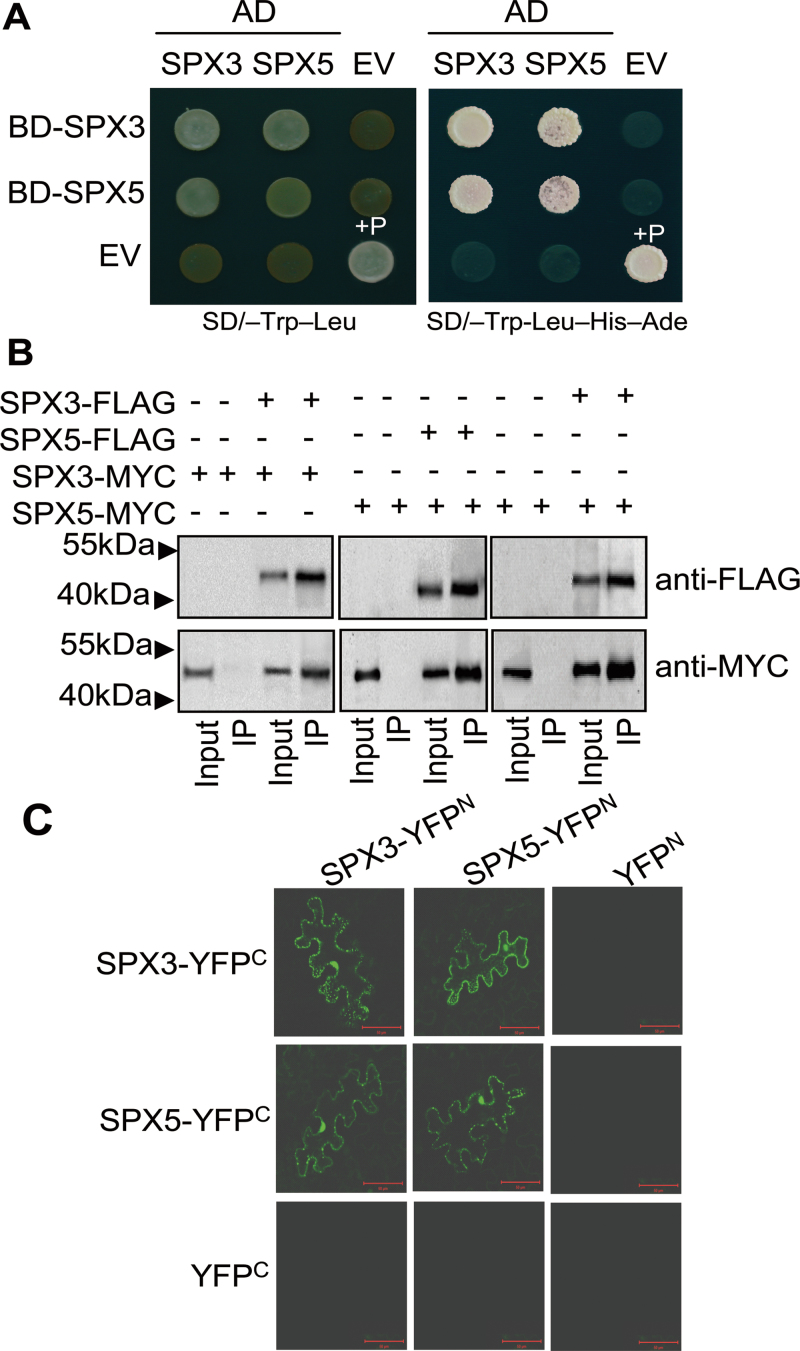
*In vitro* and *in vivo* protein interaction analyses between SPX3 and SPX5. (A) Yeast two-hybrid assays indicate that SPX3/5 can form homodimers and heterodimers in yeast cells. pGADT7-SPX3/5 (AD-SPX3/5); pGBKT7-SPX3/5 (BD-SPX3/5); EV, the empty vector; +P, p53:T7 as a positive control. (B) Coimmunoprecipitation assays indicate the interactions between SPX3 and SPX5, and with themselves *in vivo*. Tobacco leaves were infiltrated with *Agrobacterium* harbouring *35S-SPX3-MYC*/*35S-SPX3-FLAG*, *35S-SPX5-MYC*/*35S-SPX5-FLAG*, and *35S-SPX5-MYC*/*35S-SPX3-FLAG* for transient expression. Protein extracts (Input) were immunoprecipitated (IP) with anti-FLAG antibody and resolved by SDS-PAGE. (C) BiFC analysis for the interactions in *Agrobacterium*-infiltrated tobacco leaves. Coexpression of *SPX3-YFP* ^*C*^ */SPX3-YFP* ^*N*^ *, SPX5-YFP* ^*C*^ */SPX5-YFN* ^*C*^, *SPX3-YFP* ^*C*^ */SPX5-YFP* ^*N*^, and *SPX5-YFP* ^*C*^ */SPX3-YFN* ^*C*^ in tobacco leaves showed BiFC fluorescence in the nucleus and cytoplasm. Coexpression of *SPX3-YFP* ^*C*^ */YFP* ^*N*^, *SPX5-YFP* ^*C*^ */YFP* ^*N*^, and *YFP* ^*C*^ *-YFP* ^*N*^ were used as negative controls; bar, 50 µm.

The redundancy and the overall similar tissue expression patterns of SPX3/5 led to the hypothesis that the two proteins might interact with each other. Yeast two-hybrid assays were performed to explore this idea. The results showed that SPX3/5 can form homodimers as well as heterodimers in yeast cells (Fig. 8A). To verify the interactions, this work performed coimmunoprecipitation assays using transiently expressed proteins of SPX3-MYC or SPX3-FLAG, and SPX5-MYC or SPX5-FLAG in tobacco leaves. The coimmunoprecipitation assays for the combinations of fusions showed results consistent with those in yeast cells (Fig. 8B). *In vivo* interactions between SPX3 and SPX5, or with themselves, were also indicated by BiFC assays in tobacco leaves (Fig. 8C). Taken together, the findings suggest that SPX3/5 can form homodimers and heterodimers *in vitro* and *in vivo*, which supports the concept of their redundant effect, and implies a sophisticated regulation in modulating Pi homeostasis and signalling.

## Discussion

This work reports that SPX3/5 evolved in cereal crops redundantly modulate Pi homeostasis and signalling. The data indicate that the SPX proteins act as repressors of PHR2, the rice orthologue of AtPHR1, which is a central regulator of Pi signalling and homeostasis (Rubio *et al.*, 2001; Bustos *et al.*, 2010). A previous report described SPX1 as repressing PHR2 by counteracting PHR2 function (Liu *et al.*, 2010). The functional similarity of SPX3/5 to SPX1 found in this study indicates an integrated negative regulation system of Pi signalling and homeostasis by SPX proteins in crops. The role of SPX1 in negative regulation of PHR2 has been reported (Liu *et al.*, 2010), and the current findings provide evidence that the negative regulation of Pi homeostasis and signalling involves different SPX proteins. The evolution of the additional redundant paralogous SPX genes in crops implies a more controllable regulation system for crops to adapt to environmental Pi variations.

### *SPX3* and *SPX5* are paralogous genes evolved in cereal crops

Four and six SPX proteins have been identified in *Arabidopsis* and rice, respectively, designated as AtSPX1–AtSPX4 and OsSPX1–OsSPX6 (SPX1–6) (Duan *et al.*, 2008; Wang *et al.*, 2009b). These SPX proteins are grouped into three evolutionary clades (Secco *et al.*, 2012). This study cloned the full-length *SPX5* and *SPX6* through RACE assays and analysed the phylogenetic relationship of the six SPX proteins in dicotyledonous and cereal monocotyledonous plants to investigate whether the two additional SPX proteins in rice had evolved in other crops. The rooted phylogenetic tree generated from alignments of the SPXs in the tested plants and the ancestral genome *C. reinhardtii* indicated that *SPX3*, *SPX5*, and *SPX6* are paralogous genes in cereal crops (Fig. 1). There were two copies of *SPX3* and *SPX4* and three *SPX1* copies in banana. Three rounds of whole-genome duplications in the *Musa* lineage, independently of those previously described in the Poales lineage, appear to have occurred, and the earliest duplication in the *Musa* lineage is not shared with Poaceae (D’Hont *et al.*, 2012). The three copies of paralogous SPX1 and two copies of SPX3 and SPX4 in the *Musa* imply these independent whole-genome duplications. The classical models predict that the most likely fate of duplicate gene pairs is degeneration into a pseudogene or loss from the genome because of the vagaries of chromosomal remodelling, locus deletion, or point mutation (in a process known as nonfunctionalization; Prince and Pickett, 2002). The results indicate that the paralogous genes *SPX3*, *SPX5*, and *SPX6* might have been maintained in cereals during their whole-genome duplication (Tang *et al.*, 2010; Wang *et al.*, 2011) but not in dicotyledonous plants. The redundancy of the paralogous SPX3/5 as repressors of the central regulator of PHR2 of Pi homeostasis and signalling presented in this study implies that a more controllable response to Pi starvation evolved in crops.

### SPX3/5 redundantly modulate root-to-shoot Pi translocation

Both Pi inadequacy and Pi excess are detrimental to plant growth. Therefore, Pi homeostasis is required for plant growth and development, and the regulatory mechanism of Pi homeostasis is conserved in many plant species (Chiou and Lin, 2011). The current data showed that SPX3/5 redundantly modulate root-to-shoot Pi translocation, as the following evidence supports: (1) *spx3/RiSPX5* plants, but not the *spx3* mutant or the plants with knockdown of *SPX5*, displayed increased shoot Pi accumulation, higher Pi uptake activity, and Pi translocation from root-to-shoot; (2) overexpression of SPX3/5 suppressed root-to-shoot translocation of Pi; and (3) SPX3/5 can form homodimers and heterodimers (Figs. 4, 6, and 8).

SPX3/5 were expressed in root epidermal cells, including epidermis, exoepidermis, and the sclerenchyma layer (Figs. 2 and 3). From this expression pattern, it was expected that SPX3/5 would be involved in Pi uptake. The transgenic plants with overexpressed SPX3/5, however, showed decreased root-to-shoot Pi translocation, indicating a main function of SPX3 and SPX5 in Pi translocation from root to shoot. The genetic interaction analysis indicated that overexpression of SPX3 and SPX5 completely rescued the excessive shoot Pi accumulation and the upregulation of PSI genes driven by overexpression of PHR2 (Fig. 7). PHR2 directly upregulates PT2, and mutation of PT2 almost completely rescues the excessive shoot Pi accumulation caused by overexpression of PHR2 (Liu *et al.*, 2010), indicating that the PHR2-driven excessive accumulation of shoot Pi is largely attributable to upregulation of PT2. Tissue expression patterns show that PT2 is predominantly expressed in root stele and the sclerenchyma layer and in leaf mesophyll and phloem in the vascular bundle (Ai *et al.*, 2009). The overlapped expression patterns of SPX3/5 and PT2 and the negative regulation of transcript accumulation levels of *PT2* by SPX3/5 may, at least partially, explain the function of SPX3 and SPX5 in Pi translocation.

The central pathway of Pi signalling and homeostasis under the control of the partially redundant transcription factors AtPHR1/AtPHL1 (PHR1-like1) is conserved in plants (Rubio *et al.*, 2001; Duan *et al.*, 2008; Valdés-López *et al.*, 2008; Zhou *et al.*, 2008; Wang *et al*., 2009a, 2013; Bustos *et al.*, 2010; Ren *et al.*, 2012). The non-coding RNAs *IPS1* and *miR399* are specifically responsive to Pi starvation as the targets of AtPHR1. *miR399* targets the mRNA of *PHO2* and directs the cleavage of *PHO2* mRNA, which encodes a ubiquitin-conjugating E2 enzyme (Aung *et al.*, 2006; Bari *et al.*, 2006). Reciprocally, *IPS1* represses *miR399* through a mechanism termed ‘target mimicry’, interfering with miR399 targeting of *PHO2* mRNA (Franco-Zorrilla *et al.*, 2007). Loss of function of PHO2 leads to excessive shoot Pi accumulation. PHO1 mediates Pi loading to the xylem and consequently controls the root-to-shoot Pi translocation (Poirier *et al.*, 1991; Hamburger *et al.*, 2002; Secco *et al.*, 2010). Recently, PHO2-dependent degradation of PHO1 as a function of cellular Pi concentration was described (Liu *et al.*, 2012). miR399, *PHO2*, and *PHO1* form a branch of the Pi-signalling network downstream of AtPHR1. The current data showed that transcript accumulation levels of *PHO2* are significantly suppressed in *spx3/RiSPX5* plants and conversely in plants with overexpressed SPX3/5 (Figs. 5 and 6). Thus, the alteration of transcript accumulation levels of *PHO2* regulated by SPX3/5 through their negative effect on PHR2 can be inferred. This finding increases the understanding of SPX domain proteins in regulated Pi homeostasis and signalling under the control of PHR2 in rice.

### SPX3 and SPX5 are involved in fine-tuning Pi signalling

When plants are exposed to Pi starvation, a large proportion of the transcriptional activation and repression as an integral part of the Pi-starvation response is under the control of the partially redundant transcription factors AtPHR1 and AtPHL1 (PHR1-like1) (Bustos *et al.*, 2010). This genome-wide reprogramming of genes has been suggested to inhibit plant growth, but not as a direct consequence of Pi deficiency (Rouached *et al.*, 2011). The current data showed that *SPX3/5* are positively responsive to Pi starvation largely under the control of PHR2 and that their proteins accumulated under Pi starvation (Supplementary Figs. S1 and S4). Overexpression of SPX3/5 and genetic interaction analysis indicated that SPX3/5 are repressors of PHR2 function (Figs. 6 and 7). Thus, it was expected that double repression of SPX3/5 would enhance Pi-starvation signalling. Under Pi-supplied conditions, significant upregulation of PSI genes downstream of PHR2 in *spx3/RiSPX5* plants was found (Fig. 5); however, the expected enhanced Pi signalling under Pi starvation was not. *SPX6*, as the paralogous gene of *SPX3/5*, may play a compensatory role (Figs. 5 and 6), and *SPX1* RNAi lines are correlated with increased transcript accumulation levels of some PSI genes (Wang *et al.*, 2009a), so it is speculated that SPX3/5 might be involved in a fine-tuned regulation of Pi signalling together with other SPX proteins. This reasoning is supported by the results that Pi-starvation-accumulated *SPX3/5* transcripts played a negative role in reduction of Pi-starvation signalling in a time course of Pi recovery after Pi starvation (Fig. 5). The Pi-starvation-induced transcripts of *SPX3/5* were rapidly suppressed by Pi recovery over 12h while the induced protein levels were maintained for a longer time after Pi recovery (Supplementary Fig. S4). This result suggests that, following reduction of Pi-starvation-induced expression of *SPX3/5*, the Pi-starvation-accumulated SPX3/5 proteins still played a negative role in Pi-starvation signalling. This negative role should be beneficial to plants in recovering Pi homeostasis after Pi starvation.

## Supplementary material

Supplementary data are available at *JXB* online.

Supplementary Fig. S1. *SPX3/5/6* are specifically induced by Pi starvation largely under the control of PHR2.

Supplementary Fig. S2. Subcellular localization of SPX3/5.

Supplementary Fig. S3. Isolating the *spx3* mutant and development of the transgenic plants.

Supplementary Fig. S4. Time course of transcript and protein levels of SPX3/5 during Pi supply after Pi starvation.

Supplementary Fig. S5. Biomass of shoots and roots of SPX3/5-overexpressed plants.

Supplementary Fig. S6. qRT-PCR analysis for transcript accumulation levels of *PHR2* and *SPX3* in OxPHR2/OxSPX3-1 and of *PHR2* and *SPX5* in OxPHR2/OxSPX5-1 plants.

Supplementary Table S1. Primers used in this study.

Supplementary Data
