# Supplementary material for: The paralogous SPX3 and SPX5 genes redundantly modulate Pi homeostasis in rice
Source: J Exp Bot. 2013 Dec 24;65(3):859–70. doi: 10.1093/jxb/ert424 (PMC3924727; doi:10.1093/jxb/ert424)
Supplement: Supplementary Data [file supp_65_3_859__index.html]

The paralogous SPX3 and SPX5 genes redundantly modulate Pi homeostasis in rice — The paralogous SPX3 and SPX5 genes redundantly modulate Pi homeostasis in rice — Supplementary Data 

# The paralogous *SPX3* and *SPX5* genes redundantly modulate Pi homeostasis in rice

## Supplementary Data

Data files

**Files in this Data Supplement:**

- Supplementary Data - Supplementary Data
