# Supplementary material for: The paralogous SPX3 and SPX5 genes redundantly modulate Pi homeostasis in rice
Source: J Exp Bot. 2013 Dec 24;65(3):859–70. doi: 10.1093/jxb/ert424 (PMC3924727; doi:10.1093/jxb/ert424)
Supplement: Supplementary Data [file supp_ert424_jexbot109173_file001.pdf]

SUPPLEMENTAL DATA

Supplemental Methods

Subcellular localization in protoplast

The plasmid *35S-SPX3-GFP* for subcellular localization analysis had been constructed (Z. Wang *et al*., 2009). To construct the subcellular localization plasmid *35S-SPX5-GFP*, the full-length CDS of SPX5 was cloned with specific template by SPX5-GFP-F and SPX5-GFP-R, and were inserted into pCAMBIA 1302 (Z. Wang *et al*., 2009) for fusion with the reporter gene. For subcellular colocalization experiments, the ER fluorescent marker (35S-PHF1-mCherry) used has been previously reported (Chen *et al*., 2011), and 35S-CHL1-mCherry (the PM fluorescent marker) (Ho *et al*., 2009) construct was performed as described by Chen *et al*. (2011). The rice protoplast transfection and fluorescence microscopy followed previously described procedures (Chen *et al*., 2011). The sequences of the primers used for cloning are listed in Supplemental Table S1.

Subcellular localization in transgenic plants harboring *SPX3/5pro-SPX3/5-GFP* fusions

To construct the SPX3p-SPX3-GFP and SPX5p-SPX5-GFP vectors, the Nipponbare genomic DNA fragments containing the entire SPXs coding regions and 2.9-kb upstream sequences were amplified with primer pairs *SPX3pro-SPX3-GFP-F/SPX3pro-SPX3-GFP-R* and *SPX5pro-SPX5-GFP-F/SPX5pro-SPX5-GFP-R*, and generated using pGWB4 as the Gateway destination vector, respectively (Nakagawa *et al*., 2007). The transgenic plants of 10-day old were treated without Pi for 5 days. The GFP images in rice root epidermis were detected by confocal microscope.

Immunoblot analysis

For extraction of total protein, the samples were ground in liquid nitrogen and dissolved in protein lysis buffer (2% SDS, 60 mM Tris-HCl [pH8.5], 2.5% glycerol, 0.13 mM EDTA, 1mM PMSF, 100 µM MG132 [Sigma-Aldrich] and 1× complete protease inhibitor cocktail [Roche]). Total protein (30 to 50 µg) of each sample was loaded onto 12% Bis-Tris SDS-PAGE gels (NuPAGE System) and

transferred to polyvinylidene difluoride membranes. The membrane was blocked with 1% BSA in 1×TBS solution with 0.2% Tween 20 (TBST)， at room temperature for more than 30 min and hybridized with primary antibody (1:250) (rabbit anti-GFP polyclonal, Sigma) in 1×TBST for overnight at 4°C. After three washes in TBST for 5min, membranes were developed using peroxidase-conjugated secondary antibody (1:2000) (anti-mouse IgG, Sigma), and antigen protein was detected by chemiluminescence using an ECL-detecting reagent according to the manufacturer's protocol (Thermo Scientific).

Southern blot analysis

Genomic DNA was isolated using the SDS method (Murray and Thompson, 1980) and was digested with *Hind* III. DNA (5 μg) from each sample was separated on a 0.8% agarose gel and transferred to Hybond-N^+ Nylon membranes (Amersham, Pharmacia, UK). The Southern blot hybridization was performed as described previously (Zhou *et al*., 2008).

Supplemental References

Wang ZY, Hu H, Huang HJ, Duan K, Wu ZC, Wu P. 2009. Regulation of OsSPX1 and OsSPX3 on Expression of OsSPX Domain Genes and Pi-starvation Signaling in Rice. *Journal of Integrative Plant Biology* **51,** 663–674.

Chen J, Liu Y, Ni J, Wang Y, Bai Y, Shi J, Gan J, Wu Z, Wu P. 2011. OsPHF1 regulates the plasma membrane localization of low- and high-affinity inorganic phosphate transporters and determines inorganic phosphate uptake and translocation in rice. *Plant Physiology* **157,** 269-278.

Ho CH, Lin SH, Hu HC, Tsay YF. 2009. CHL1 Functions as a Nitrate Sensor in Plants. *Cell* **138,** 1184-1194.

Nakagawa T, Kurose T, Hino T, Tanaka K, Kawamukai M, Niwa Y, Toyooka K, Matsuoka K, Jinbo T, Kimura T. 2007. Development of series of gateway binary vectors, pGWBs, for realizing efficient construction of fusion genes for plant transformation. *Journal of Bioscience and Bioengineering* **104,**

34-41.

Murray MG, Thompson WF. 1980. Rapid isolation of high molecular weight plant DNA. *Nucleic Acids Research* **8,** 4321–4325.

Zhou J, Jiao F, Wu Z, Li Y, Wang X, He X, Zhong W, Wu P. 2008. *OsPHR2* is involved in phosphate-starvation signaling and excessive phosphate accumulation in shoots of plants. *Plant Physiology* **146,** 1673-1686.

Supplementary Material Legends

Supplementary Fig. S1. *SPX3/5/6* are specifically induced by Pi-starvation largely under the control of PHR2. (A–C) qRT-PCR analysis indicates that the specifically induced expressions of *SPX3*, *SPX5* and *SPX6* by Pi starvation. Total RNA was extracted from shoots and roots of the seedlings (Nipponbare) grown in normal nutrient solution for 20 days followed by treatment with (CK) or without Pi (P), nitrogen (N), potassium (K), sulfur (S), calcium (Ca), magnesium (Mg) or iron (Fe) for 7 days. (D and E) qRT-PCR analysis indicates that the Pi-starvation induced expressions of *SPX3*, *SPX5* and *SPX6* are remarkably reduced in *phr2* mutant in both shoot and root. The total RNA extraction and Pi-starvation treatment are the same as in (A–C). WT: wild type (ZH11). +P: Pi supplied solution (200 μM Pi); -P: without Pi supplied solution. Values represent means ± SD of three biological replicates. Data significantly different from the corresponding controls are indicated (the nutrient sufficient versus the nutrient deficient, ** P<0.01; *phr2* mutant versus the wild type, ++ P<0.01; Student's *t* test).

Supplementary Fig. S2. Subcellular localization of SPX3/5. (A) Subcellular localization analysis of SPX3/5 in rice protoplast cells. Localization of SPX3/5 in nucleus and cytoplasm is indicated by the plasma membrane (PM) marker (CHL), and endoplasmic reticulum (ER) marker (PHF1), respectively. Bar=10 μm. (B) Expressions of fused proteins of SPX3pro-SPX3-GFP and SPX5pro-SPX5-GFP, respectively, in the transgenic plants. Bar=50 μm. The GFP images in rice root epidermis were detected by confocal microscope.

Supplementary Fig. S3. Isolating *spx3* mutant and development of transgenic plants. (A–C) PCR and RT-PCR analyses indicate the T-DNA insertion site in the second exon of *SPX3* and absent transcript of *SPX3* in *spx3* mutant. (D–F) qRT-PCR analysis for expression of *SPX3* or *SPX5* in the transgenic lines. Values represent means ±SD of three replicates. The independent lines (*RiSPX5-1*, *RiSPX5-3*, *OxSPX3-1*, *OxSPX3-2*, *OxSPX5-1*, and *OxSPX5-2*) were used for development of T_2 lines. (G) Southern blotting analysis of *RiSPX5* plants. (H) Southern blotting analysis for expressions of *SPX3* and *SPX5* (*OxSPX3* and *OxSPX5)* in the transgenic plants with overexpressions of *SPX3/5*, respectively.

Supplementary Fig. S4. The transcript and protein levels of SPX3/5 under turnover of Pi conditions. (A) qRT-PCR analysis for expressions of *SPX3/5* in roots of the wild type (WT) plants in a time-course of Pi recovery after Pi-starvation for 10 days. Values represent means ±SD of three biological replicates. (B and C) The levels of SPX3/5 proteins in roots of the transgenic plants harboring *SPX3pro-SPX3-GFP* (B) or *SPX5pro-SPX5-GFP* (C) fusion. The treatment of the transgenic plants is the same as in (A). The CBB-stained bands are shown as loading control.

Supplementary Fig. S5. Biomass of shoots and roots of SPX3-/5-overexpressed plants. Dried biomass of 30d-old wild type (WT) and *SPX3-/5*-overexpressed plants (*OxSPX3* and *OxSPX5*) grown in solution cultures with 200 μM Pi (A) and 20 μM Pi (B) conditions. Values represent means ±SD of five plants. Data significantly different from the corresponding WT controls are indicated (** P<0.01, Student's t test).

Figure S6. qRT-PCR analysis for transcript accumulation levels of *PHR2* and *SPX3* in *OxPHR2/OxSPX3-1* and of *PHR2* and *SPX5* in *OxPHR2/OxSPX5-1*.

Supplementary Table S1. The primers used in this study

Supplementary Table S1. The primers used in this study

Primer Name and Description	Sequence (5′—3′)
For RACE-PCR:	
SPX5-3P1	CAGTCCGTCCAGCCGCCGC
SPX5-3P2	CGCCGTCGTCGCCGCTCATC
SPX6-3P1	AGACGGAGACGGTGTCGCGGATGGTG
SPX6-3P2	GACTCCGACTGGCTCCGCT
B25	GACTCGAGTCGACATCGAT (T)$_{18}$
B26	GACTCGAGTCGACATCGAT
SPX5-5P1	ACGACGACTGGATCCTCTCCTG
SPX5-5P2	GGAAGAAGGCGTTGAACTTGTC
SPX6-5P1	CACCATCCGCGACACCGTCTCCGTCT
SPX6-5P2	AGTCCACGATCTCCCGCCGGATTCC
AAP	GGCCACGCGTCGACTAGTAC (G)$_{14}$
AUAP	GGCCACGCGTCGACTAGTAC
For plasmid constructs:	
SPX5-p-g-GFP-F	CACCCGCTGTCAAGTCGCCACATGGCAT
SPX5-p-g-GFP-R	CGTGGGGATGATGAGCGGCGACG
SPX3-p-g-GFP-F	CACCGTCCACGTGTAGGGATTAGGGAC
SPX3-p-g-GFP-R	GGCATAAAAAAACTGTAAACTTGGAATT
SPX5-GFP-F	GCGCAGATCTATGAAGTTCGGGAAGCGGCTGAAG<br>*Bgl*II
SPX5-GFP-R	GCGCACTAGTCGTGGGGATGATGAGCGGCGACG<br>*Spe*I
SPX3-GUS-F	TCTAGAGGATCCACGGTACCCCGTCCACGTGTAGGG<br>ATTAGGGAC
SPX3-GUS-R	CTCAGATCTACCATGGTACCGGCATAAAAAAACTGT<br>AAACTTGGA
SPX5-GUS-F	TCTAGAGGATCCACGGTACCCCTCCTCTATGGACGA<br>TACAAACGG
SPX5-GUS-R	CTCAGATCTACCATGGTACCCGTGGGGATGATGAGC<br>GGCGACGAC
CHL1-mCherry-F	GCGC*GTCGAC*ATGTCTCTTCCTGAAACTAAATCTGAT<br>*Sal*I
CHL1-mCherry-R	GCGCACTAGTTCAATGACCCATTGGAATACTCG<br>*Spe*I
SPX3 insitu-F	GCTGCCGTTCATCGAGAAGGTGC
SPX3 insitu-T7-R	GAATTGTAATACGACTCACTATAGGG<br>CTCTACCACGGCATATGTGAGTTTAC
SPX3 insitu-SP6-F	GAATTGATTTAGGTGACACTATAG<br>GCTGCCGTTCATCGAGAAGGTGC
SPX3 insitu-R	CTCTACCACGGCATATGTGAGTTTAC
SPX5 insitu-F	GTGCTCCGCCTCCCCGTCATCG
SPX5 insitu-T7-R	GAATTGTAATACGACTCACTATAGGGCAAGAACCATT

	GGTATTGATC
SPX5 insitu-SP6-F	GAATTGATTTAGGTGACACTATAGGCTTGTTCGAGAA TGTGAGGC
SPX5 insitu-R	CAAGAACCATTGGTATTGATC
SPX3-Ov-F	GCGGGTACCATGAAGTTTGGGAAGAGGCTG
	*Kpn*I
SPX3-Ov-R	GCGTCTAGATCTCTACCACGGCATATGTGAG
	*Xba*I
SPX5-Ov-F	GCGCCCCGGGATGAAGTTCGGGAAGCGGCTGAAG
	*Sma*I
SPX5-Ov-F	GCGCCCCGGGCCATCAAGAACCATTGGTATTGATCTC
	*Sma*I
SPX5-RNAi-F	CGCCGTCGTCGCCGCTCATC
SPX5-RNAi-R	CAAGAACCATTGGTATTGATC
SPX3-YF	AATCATATGAAGTTTGGGAAGAGGCT
	*Nde*I
SPX3-YR	ATTGGATCCTTAGGCATAAAAAAACTGTA
	*Bam*HI
SPX5-YF	GGACATATGAAGTTCGGGAAGCGGCT
	*Nde*I
SPX5-YR	AATGAATTCCTACGTGGGGATGATGAGCG
	*Eco*RI
SPX3-2YN-F	CCCTTAATTAACATGAAGTTTGGGAAGAGGCTGAAG AAGC
	*Pac*I
SPX3-2YN-R	AAAGGCGCGCCCGGCATAAAAAAACTGTAAACTTGG AATT
	*Asc*I
SPX5-2YN-F	CCCTTAATTAACATGAAGTTCGGGAAGCGGCTGAAG AGG
	*Pac*I
SPX5-2YN-R	AAAGGCGCGCCCCGTGGGGATGATGAGCGGCGACGA CGG
	*Asc*I
SPX3-Myc-F	AAAGGTACCATGAAGTTTGGGAAGAGGCTGAAG
	*Kpn*I
SPX3-Myc-R	AAAGGATCCCGGCATAAAAAAACTGTAAACTTGG
	*Bam*H1
SPX5-Myc-F	AAAGGTACCATGAAGTTCGGGAAGCGGCTGAAGAG
	*Kpn*I
SPX5-Myc-R	AAAGGTACCCCGTGGGGATGATGAGCGGCGACGACG
	*Kpn*I
SPX3-FLAG-F	CCCTCTAGAATGAAGTTTGGGAAGAGGCTGAAG
	*Xba*I
SPX3-FLAG-R	CCCTCTAGAGGCATAAAAAAACTGTAAACTTGG
	*Xba*I

SPX5-FLAG-F	CCCTCTAGAATGAAGTTCGGGAAGCGGCTGAAG
	*Xba*I
SPX5-FLAG-R	CCCTCTAGACGTGGGGATGATGAGCGGCGACG
	*Xba*I
Identification of T-DNA mutant:	
Osphr2-P1	CGCCTCTGCCTGCTATACCA
Osphr2-P2	CTAAGCATGCCCAATTGAAA
Osphr2-P3	AATCCAGATCCCCCGAATTA
CAM6	CGCTCATGTGTTGAGCATAT
OsSPX3-RT3-F	GGAGATGGTGCTGCTGCTTA
OsSPX3-T-R	AACACAGCCTAGTCACTTACCGAT
OsActin-RT-F	GGAACTGGTATGGTCAAGGC
OsActin-RT-R	AGTCTCATGGATACCCGCAG
OsSPX3-RT3-F	GGAGATGGTGCTGCTGCTTA
OsSPX3-RT3-R	GGAAGATGCCCTGCTGGTCT
Reverse-transcribe specific primers and qRT-PCR primers:	
Osa-miR399a/b/c/d/i-RT	GTTGGCTCTGGTGCAGGGTCCGAGGTATTCGCACCA GAGCCAACCAGGGC
Osa-miR399e/f/g/h-RT	GTTGGCTCTGGTGCAGGGTCCGAGGTATTCGCACCA GAGCCAACCTGGGC
Osa-miR399j-RT	GTTGGCTCTGGTGCAGGGTCCGAGGTATTCGCACCA GAGCCAACTAGGGC
Osa-miR399k-RT	GTTGGCTCTGGTGCAGGGTCCGAGGTATTCGCACCA GAGCCAACCGGGGC
OsActin-qRT-F	CAACACCCCTGCTATGTACG
OsActin-qRT-R	CATCACCAGAGTCCAACACAA
OsSPX3-qRT-F	TGCAGTCCATCCGATCCG
OsSPX3-qRT-R	ATGTGTATGTATGTTCTCTACCACG
OsSPX5-qRT-F	CGACGAGCTGCAACATT
OsSPX5-qRT-R	CAAGAACCATTGGTATTGATC
OsIPS1-qRT-F	AAGGGCAGGGCACACTCCACATTATC
OsIPS1-qRT-R	ATTAGAGCAAGGACCGAAACACAAAC
OsmiR399-d/j/k-F	CGGCGGTGCCAAAGGAGAGTT
OsmiR399-a/b/c-F	CGGCGGTGCCAAAGGAGAATT
OsmiR399-e/f/g-F	CGGCGGTGCCAAAGGAGAATT
OsmiR399-h-F	CGGCGGTGCCAAAGGAGACTT
OsmiR399-i-F	CGGCGGTGCCAAAGGAGAGCT
OsmiR827-F	CGGCGGTTAGATGACCATCAG
Universal Reverse primer	GTGCAGGGTCCGAGGT
OsPHO2-qRT-F	CGAGAATTTTGTCAAGGAGCA
OsPHO2-qRT-R	TCACGAGCATGTCCAACAA
OsPT2-qRT-F	GTGGTCGAGAGCCAGGTG
OsPT2-qRT-R	TTCATGAACTGCCGTGAGAA

OsSPX3-qRT-T-F	GACAGGATCAACGCCTTCTT
OsSPX3-qRT-T-R	CCACCTTCTCCACTGTCTCC
OsSPX5-qRT-Ri-F	CATCTTCCGCAACACCGTCG
OsSPX5-qRT-Ri-R	GCGGCTGGACGGACTGGATG
OsSPX6-qRT-F	TCTGCGCTGCGAAATCTG
OsSPX6-qRT-R	TTGAAAGCCAAAACACGTATG
OsPAP10-qRT-F	ATACTGGCAGCCGACGGATGA
OsPAP10-qRT-R	GAGGGAGCTGGAGCGGAGAA
OsSQD2-qRT-F	CTGAAAACGGTAATGGATAGG
OsSQD2-qRT-R	AACAACAACAGCACGAGC

Figure S1

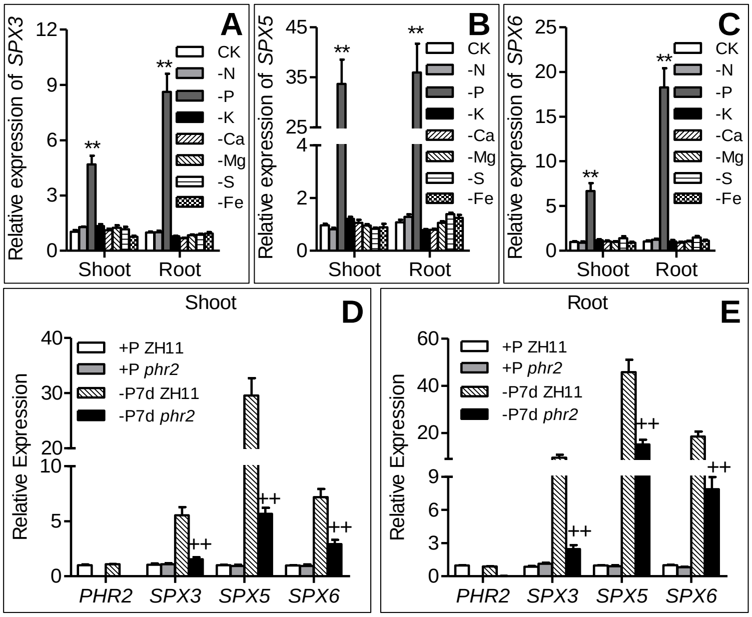

A
Relative expression of SPX3
CK
-N
-P
-K
-Ca
-Mg
-S
-Fe
Shoot
Root
B
Relative expression of SPX5
C
Relative expression of SPX6
D
Shoot
Relative Expression
+P ZH11
+P phr2
-P7d ZH11
-P7d phr2
PHR2
SPX3
SPX5
SPX6
E
Root

Figure S2

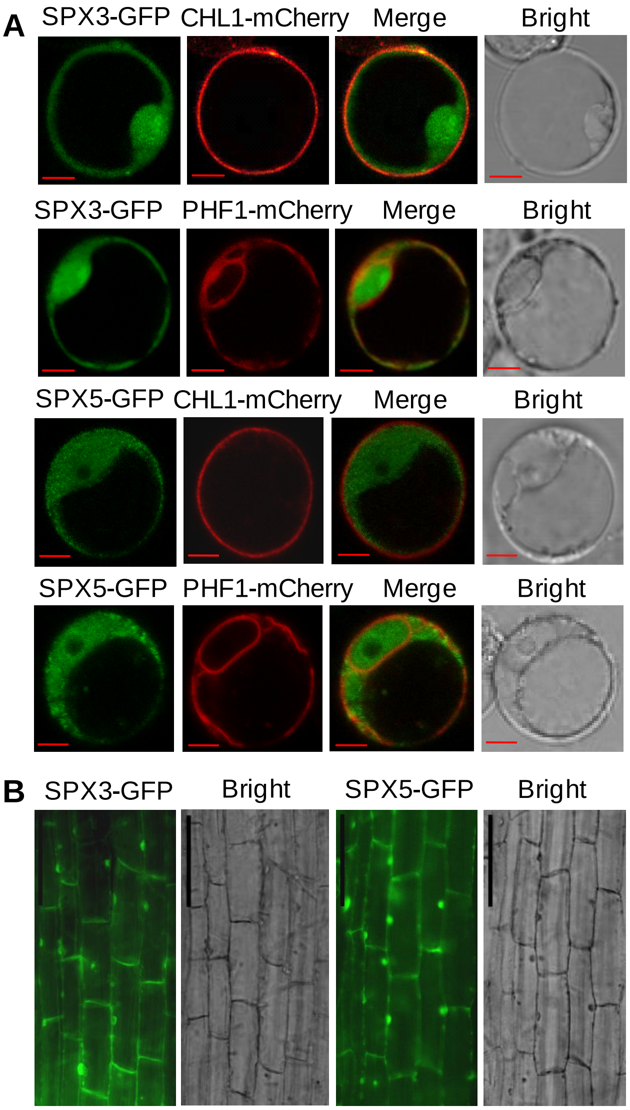
A
SPX3-GFP
CHL1-mCherry
Merge
Bright
SPX3-GFP
PHF1-mCherry
Merge
Bright
SPX5-GFP
CHL1-mCherry
Merge
Bright
SPX5-GFP
PHF1-mCherry
Merge
Bright
B
SPX3-GFP
Bright
SPX5-GFP
Bright

Figure S3

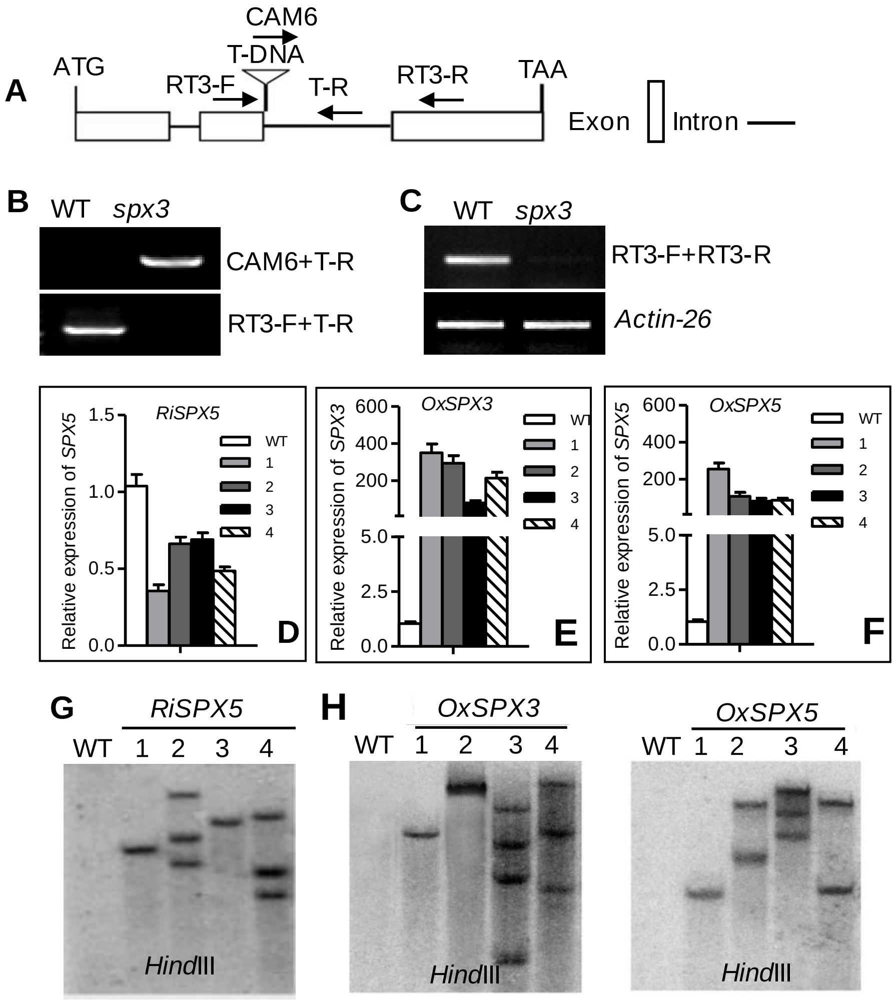
A
CAM6
T-DNA
ATG
RT3-F
T-R
RT3-R
TAA
Exon
Intron
B
WT spx3
CAM6+T-R
RT3-F+T-R
C
WT spx3
RT3-F+RT3-R
Actin-26
D
RiSPX5
Relative expression of SPX5
WT
1
2
3
4
E
OxSPX3
Relative expression of SPX3
F
OxSPX5
Relative expression of SPX5
G
RiSPX5
WT 1 2 3 4
HindIII
H
OxSPX3
WT 1 2 3 4
HindIII
OxSPX5
WT 1 2 3 4
HindIII

Figure S4

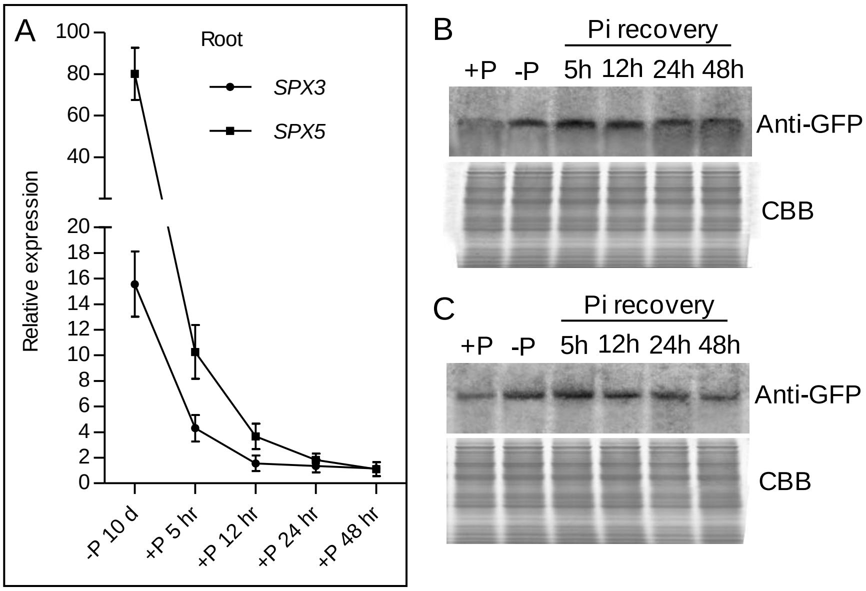
A
Root
SPX3
SPX5
Relative expression
100
80
60
40
20
18
16
14
12
10
8
6
4
2
0
-P 10 d
+P 5 hr
+P 12 hr
+P 24 hr
+P 48 hr
B
Pi recovery
+P -P 5h 12h 24h 48h
Anti-GFP
CBB
C
Pi recovery
+P -P 5h 12h 24h 48h
Anti-GFP
CBB

Figure S5

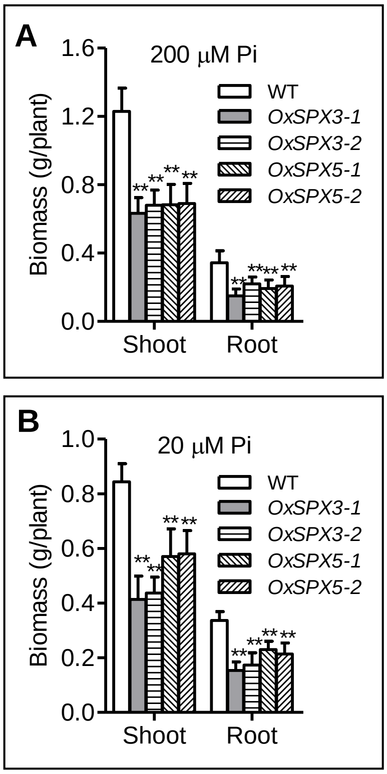
A
200 μM Pi
Biomass (g/plant)
1.6
1.2
0.8
0.4
0.0
WT
OxSPX3-1
OxSPX3-2
OxSPX5-1
OxSPX5-2
Shoot
Root
B
20 μM Pi
Biomass (g/plant)
1.0
0.8
0.6
0.4
0.2
0.0
WT
OxSPX3-1
OxSPX3-2
OxSPX5-1
OxSPX5-2
Shoot
Root

Figure S6

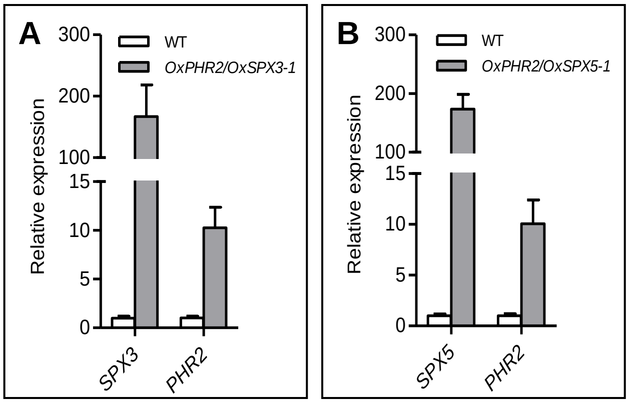

A
300
200
100
15
10
5
0
Relative expression
WT
OxPHR2/OxSPX3-1
SPX3
PHR2
B
300
200
100
15
10
5
0
Relative expression
WT
OxPHR2/OxSPX5-1
SPX5
PHR2
